# D&D and You: A Reflexive Thematic Analysis of Young Adult Players’ Experiences Exploring Identity and Mental Health Through Dungeons and Dragons

**DOI:** 10.3390/bs16061026

**Published:** 2026-06-18

**Authors:** Zoe Thomas, Abby Dunn, Aislinn D. Gomez Bergin, Cassie M. Hazell

**Affiliations:** 1 Department of Psychological Interventions, School of Psychology, University of Surrey, Guildford GU2 7XH, UKabby.dunn@sussex.ac.uk (A.D.); 2 Responsible AI UK, School of Computer Science, University of Nottingham, Nottingham NG7 2TU, UK; aislinn.bergin@nottingham.ac.uk

**Keywords:** young adults, mental health, Dungeons and Dragons, identity, tabletop roleplaying game, qualitative

## Abstract

Dungeons and Dragons (D&D) is a collaborative roleplaying game that is associated with social and emotional benefits for young adults (YAs). Research has not addressed how YAs’ understanding of identity and mental health is explored through D&D. This research explored the impact that playing D&D has on YAs’ understanding of their identity and how this relates to their mental health. Eleven YAs (aged 18–25) were interviewed about their experiences of playing D&D. Their interviews were analysed using Reflexive Thematic Analysis. Four main themes were identified: D&D as a safer space; D&D for coping; D&D for exploration; and D&D for growth. The findings demonstrate that YAs use D&D to navigate experiences such as social and emotional difficulties and their evolving sense of self. D&D helped YAs to manage their current circumstances as well as to look to the future. These findings highlight the positive impact D&D has on identity exploration and mental health for YAs. Playing D&D was perceived by participants as improving their wellbeing, relationships, and occupations at a critical time developmentally when they are developing their sense of self. Consideration of how D&D may be incorporated into existing intervention approaches is discussed, including implications for further research.

## 1. Introduction

Dungeons and Dragons (D&D), created by Gygax and Arneson (1974), is a collaborative tabletop roleplaying game (TTRPG), facilitated by a Dungeon Master (DM), sometimes also known as a Games Master (GM) (Silverman, 2023). Players typically create their own character to embark on fantasy adventures through an evolving curated story that is shaped by dice rolls. Games range from one session of several hours to multiple sessions over a number of weeks to years. The game is generally adaptable to the needs of the gaming group, with core guidebooks available to support both the DM/GM and players to engage with the rules of the game.

Historically, D&D was considered controversial during the “Satanic Panic”, a period of time in the 1980s when Western communities in the United States and United Kingdom became immensely concerned with indoctrination into witchcraft and demonology. This resulted in unfounded claims that playing had a negative impact on mental health (Baker et al., 2023). However, the D&D community persisted and has continued to grow over the years, with a spike in new players during the COVID-19 pandemic (Allison, 2021). D&D improved coping and increased time spent with friends and family during lockdowns (Baker et al., 2023).

There are a number of reported benefits of TTRPGs. The social aspect of these games can facilitate social connectedness (Sargent, 2014); the varied and changeable quests provide a safer space for creative problem solving (Kallam, 1984); and the inevitable in-game losses and failures improve resilience (Polkinghorne et al., 2021). A Rapid Evidence Assessment (i.e., an expedited systematic review) (Henrich & Worthington, 2023) found that D&D led to improvements in the understanding of individuals’ emotions and triggers (Rafaeli et al., 2011) and created opportunities to model and practice more effective coping in a safer and contained environment (Matthews et al., 2014). A key element of D&D is creating a personalised character through which to explore the in-game world. Many researchers argue that characters may provide a vessel for players to explore new experiences or revisit older emotions that they wish to work through (Bautista, 2022). These benefits were found in the context of recreational use of TTRPGs, but as outlined by Henrich and Worthington (2023), this learning provides a foundation for its use in the context of therapeutic interventions and systems.

TTRPGs are increasingly being explored as a means for delivering therapeutic interventions (Arenas et al., 2022) for specific populations, including families (Breen & Woodin, 2025), veterans (Battles et al., 2025), autistic people (Kohei, 2019), and those who are socially anxious (Varrette et al., 2022). These studies are at the feasibility stage of evaluation using cognitive behavioural techniques and have shown initial promising results. However, the full range of possibilities regarding the utilisation of TTRPGs is still being explored. Further work is needed to explore the potential scope of the logic model(s) that could underpin such therapies and how these can be adapted for different targets (Kilmer et al., 2023). One of the few studies that directly explored the clinical utility of D&D (Causo & Quinlan, 2021) interviewed adults recovering from mental health difficulties who played D&D to explore how D&D fit into their recovery journey. The researchers found that their responses mapped onto the Psychological Recovery Model’s five stages of recovery: moratorium, awareness, preparation, rebuilding, and growth (Andresen et al., 2003). They also found evidence of the four processes that enable recovery in players’ journeys through D&D: finding hope, re-establishment of identity, finding meaning in life, and taking responsibility for recovery. This research is limited due to its predominantly male sample but nevertheless contributes to increasing beliefs that TTRPGs can facilitate therapeutic processes. The therapeutic promise of TTRPGs has led to the production of guidance for clinicians who wish to utilise TTRPGs for therapeutic interventions (see Kilmer et al., 2023; Connell, 2023). These have been termed Therapeutically Applied Roleplaying Games (TA-RPGs—Kilmer et al., 2023), or Therapeutic Gaming (Game Therapy UK, 2025), and may be particularly effective at reaching those who do not traditionally access mental health services.

There is growing evidence suggesting that there are many mental health-related benefits to playing D&D, with key gaps that still need investigation. In particular, there is a need to understand the impacts of D&D for young adults specifically, who make up a third of D&D players (Wilde, 2023) and are at a critical timepoint in their identity development (McGoldrick & Carter, 2003). The benefits of gameplay at this age could potentially be most impactful as this represents a critical life stage for identity formation (McGoldrick & Carter, 2003) and the emergence of mental health difficulties (Granic et al., 2020). The current study therefore aimed to explore how players, specifically young adults, understand themselves and their mental health in relation to their experiences of playing D&D.

The research objectives were as follows:

(1) To explore players’ understanding of their sense of self and how this is influenced by playing D&D.
(2) To explore players’ understanding of the relationship between playing D&D and their wellbeing and mental health.
(3) To explore players’ understanding of how D&D could be used to optimise therapeutic interventions or tools.

## 2. Materials and Methods

### 2.1. Design

Online, semi-structured interviews were carried out with young adults who played D&D. Participants will be described as “players” hereafter for the purposes of this research. The research design and study materials were created through consultation with several stakeholders, including TTRPG researchers, university roleplaying society members, and other D&D players.

### 2.2. Participants

The inclusion criteria specified that participants must be: considered a young adult as defined within the National Health Service (2019) Long Term Plan (aged 18–25 years), living in the United Kingdom at the time of taking part, able to verbally communicate in English, and have a D&D character they were happy to discuss during the interview. Guidance was provided, suggesting that players should “feel familiar” with their character and have played this character for at least 6 h. Individuals could not take part if they had only engaged with D&D as a DM/GM to help homogenise the shared experience of the participants. This decision was taken because current TA-RPGs place the therapist within the DM/GM role (e.g., Kilmer et al., 2023; Connell, 2023) and their role in the game involves holding the space, which means that their opportunities for identity exploration look quite different. 

### 2.3. Recruitment

The study aimed to recruit between 10 and 20 participants as per recommendations for a Reflexive Thematic Analysis, while allowing the researchers scope to determine the final sample size based on information power in terms of the richness and complexity of the data generated (Braun & Clarke, 2021b). Convenience and snowball sampling were used to reach as many eligible players as possible. Recruitment materials were shared on social media, in community spaces, and via TTRPG groups.

### 2.4. Materials

The semi-structured interview guide was informed by previous research (Causo & Quinlan, 2021) and developed in collaboration with stakeholders. The interview was divided into three sections to explore the different aspects of D&D gameplay: (1) character creation, (2) playstyle, and (3) experience of the ruleset and tools that guide play. To help facilitate the third section of the interview, where participants considered how playing D&D could be used to support those with mental health difficulties, a short video of the researcher playing through the “Dungeon Escape” level on GatherTown was presented to participants. GatherTown (Gather Presence Inc., 2025) is an online social space that can be used for virtual conferences and workplaces. GatherTown was used to roleplay ideas that participants had for how this could be enacted to support the discussion.

Players were also invited to share a character visual. Participants were encouraged to present the visual of the character that they felt the most connected to. These visuals could be in any format and produced using any tools. Most of the visuals were illustrations that either the participants themselves or their friends had created. Some participants had used an online tool to create their design and had then downloaded the image. The character was presented using screenshare at the beginning of the interview and used as the basis for interview questions, similarly to photo elicitation methods (Harper, 2002). Using visuals in this way provides participants with alternative ways to express their experiences, which can be beneficial when discussing sensitive topics and/or where there may be power imbalances between participant and researcher (Kyololo et al., 2023).

### 2.5. Procedure

Eligible participants were provided with study information and invited to provide informed consent and demographic information. Participants were contacted to organise an interview time and date. These participants were invited to choose a pseudonym and share visuals of their chosen character that they wished to discuss during the research interview. For convenience, interviews were held on Microsoft Teams and ranged from 49 min to 1 h and 22 min (*M* = 54.68 min). Confidentiality and its limits were explained, and participants were given the opportunity to ask any additional questions prior to beginning the interview and starting the recording. After the interview, participants were sent a debrief and a £20 voucher.

### 2.6. Patient and Public Involvement (PPI)

We consulted with students from a TTRPG university society to inform the design of the research study. The feedback from these consultations helped us to define the 6 h minimum requirement of playing experience to be eligible to participate, refine the interview questions, and shape the language used in participant-facing materials when discussing mental health.

### 2.7. Ethical Considerations

This study was given favourable ethical approval by the University of Surrey Research and Integrity and Governance Office (FHMS 22-234 242 EGA) as part of the lead researchers PsychD.

### 2.8. Analysis Plan

The recordings were transcribed verbatim, removing all identifiable information. The data were analysed using Reflexive Thematic Analysis ([RTA] Braun & Clarke, 2006, 2012), following the six-phase process. Initial coding was completed and then mapped into clusters which were reviewed and refined, with the themes named. The researcher conducting the analysis completed a reflexive diary alongside the analysis, acknowledging the potential impact of their personal experience of playing D&D and professional experience as a trainee Clinical Psychologist on the interpretation of the data. This perspective meant that the researcher had insider knowledge of the game while also having a natural inclination to psychological thinking and formulation when seeking to understand the human experience. We maximised the credibility of the analysis by reflecting on emerging findings within supervision and supporting themes with verbatim quotes, while also acknowledging that from a critical realist perspective that themes cannot reflect an objective truth and instead represent an individual’s perception of reality as shaped by their experiences, culture, and language (Braun & Clarke, 2021a).

## 3. Results

A total of 74 individuals contacted the researcher via email to take part, 31 of whom completed the consent form. Thirteen individuals were invited to interview based on who completed the demographics questionnaire first while also paying attention to the diversity of the sample. Two participants withdrew from the study, leaving a final sample of 11. Recruitment ended here as the data were deemed to have sufficient depth to meet the research aims. Participants were aged between 18 and 25 years of age (*M* = 22.36 years). They had a varied range of duration playing D&D (11 months–12 years; *M* = 5.42 years), with some reporting to have been a player only (*n* = 6), whilst others also had experience as a DM (*n* = 5). Table 1 presents the participants’ experience of Dungeons and Dragons.

From the self-reported sample characteristic data, we found that the modal participant demographic was white British (*n* = 5), non-binary (*n* = 5) students who may also be employed (*n* = 6). Most participants described themselves as diagnosed with a mental health condition by a health professional and had accessed support for this (*n* = 9). Most participants also identified as having a neurodevelopmental condition, either self-diagnosed or diagnosed by a health professional (*n* = 9). Full participant demographics are presented in Table 1.

**Figure 1:**
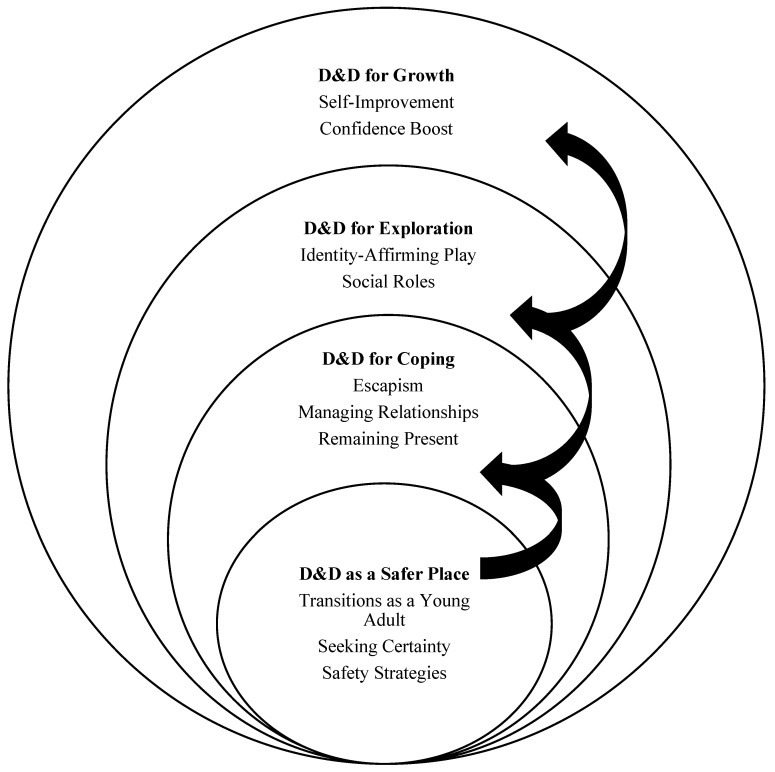
Visual of nested themes in bold that evolve from D&D as a safer place to D&D for growth, with arrows indicating directional relationships between themes. Subthemes are presented below each theme.

Four main themes and ten subthemes were developed from the data. Figure 1 presents how the themes were nested, with arrows indicating the directional relationships between the themes, presenting how they evolve from D&D as a safer place to D&D for growth.

### 3.1. D&D as a Safer Place

This initial theme identifies D&D as a place of safety for young adults during times of transition and uncertainty, whilst highlighting how safety can be created and maintained in D&D.

#### 3.1.1. Transitions as a Young Adult

Many players identified that D&D was valuable to them during significant changes in their lives such as starting university, adjusting to COVID-19 restrictions, or moving to a new country:

*I was living in a foreign country … so I joined a Facebook group for tabletop RPGs, I saw an event at a café, I went to that.*

*(Star)*

Through these experiences, players evoked a sense of trust in the D&D community to be welcoming and accepting. Additionally, players who described barriers to engaging with their usual D&D group evoked a sense of loss, expressing clear motivation to find new ways to play. These experiences seemed to describe D&D as a tool to ease challenges that players may encounter, providing a greater sense of safety despite difficulties during transitions.

#### 3.1.2. Seeking Certainty

Many players identified the sense of uncertainty they experienced during the transition into young adulthood and the ways in which D&D facilitated certainty at this time. Dungeons and Dragons appeared to provide an anchor point in their week, providing something to look forward to:

*… having one thing a week that you do really enjoy has made a lot of stuff that I’ve done a lot more manageable and feel more like I’ve got things to look forward to.*

*(Kade)*

Here Kade highlights the importance of D&D as a valued activity in their week, seemingly expressing the relief this brings during the times of high stress they experienced at university. This relief appears to be beyond that found in a recreational hobby, as several players noted that playing consistent games requires commitment from the whole group. This commitment to the game in turn appeared to be reflected in the commitment players then experience from each other:

*You feel like you’re part of something. And you feel taken care of.*

*(Stadler)*

This collective responsibility seems to build a sense of relational security between members of the gaming group, allowing them to feel valued and providing a safer space they can trust in when, by its nature, going to university or starting a new job brings many uncertainties.

#### 3.1.3. Safety Strategies

Players shared a number of different important aspects of D&D that help to maintain the games as a safer space. For example, three players described a “session zero” which has been developed by the TTRPG communities to try and provide opportunities to share expectations and concerns before beginning roleplay and starting an adventure. Here Nick describes the approach of his DM:

*This is quite a common thing that he will sit down and go OK what makes people uncomfortable? Here are potential triggering themes that are going to come up throughout the campaign.*

*(Nick)*

Players spoke about how this sense of collaborative safety and value for effective communication was essential in creating a respectful and safer atmosphere. In comparison, players who experienced poor communication and boundary setting highlighted the uncomfortable and compromised position they found themselves in.

### 3.2. D&D for Coping

The second theme, D&D for coping, considers active and passive ways in which players engage with D&D individually and socially as a tool to manage the demands of young adulthood once they experience a sense of safety within their D&D games. This theme also acknowledges the impact of this on their mental health and wellbeing.

#### 3.2.1. Escapism

Over half of the players spoke about wanting to escape the stress that young adulthood brings, such as challenges associated with higher education and difficulties in their relationships. It seemed that D&D was a valuable tool for enabling this:

*… I didn’t have to worry about the impending deadlines or the difficulties happening in my relationships, my faltering mental health … I was somewhere else … I was someone else.*

*(Bunny)*

For participants, not only did D&D provide escapism from the external factors associated with the new responsibilities of these young adults but relief through escapism from their internal experiences by embodying someone different to themselves. However, players also highlighted how over-reliance on escapism may exacerbate ongoing challenges:

*… I’m also the type of person who has taken it too far at times being like, well, I don’t need therapy because I’m not me anymore.*

*(Ashe)*

This temptation to disconnect from reality in times of stress through D&D highlights the powerful experience roleplaying can provide to players as a coping strategy. However, as noted by Ashe, awareness of how players are using D&D as a coping strategy is important to ensure that it does not prevent players from seeking alternative strategies that may feel more beneficial to them.

#### 3.2.2. Managing Relationships

Players found their characters exploring and creating relationships with other player characters and non-player characters. These bonds were developed in working towards shared goals and led to building real relationships between players in reality, whether playing online or in person. Several players reflected on the experience of choosing a family through meeting other players in D&D and the positive impact this had on their wellbeing. Additionally, one player described their experience as an adopted child. They described how their experiences with found family in D&D helped improve their connection to their siblings outside of the game:

*… just because we’re not technically blood related doesn’t mean that I can’t, you know, accept them as my brothers.*

*(Ashe)*

These experiences suggest that whilst the game may facilitate connection among a group of players, the meaning players internalise from these experiences may also support how they engage with relationships outside of D&D, which appears to positively impact their wellbeing. Positive relational experiences through D&D may even provide support in accessing further mental health support in situations where playing helps identify and share the difficulties that players experience:

*When I like opened up to them … I do panic after every session … everyone was so supportive and lovely throughout that and like we had a little celebration.*

*(Nick)*

The sense of collective care experienced by players in these supportive groups appears to facilitate trust between group members, enabling meaningful honesty and promoting self-care. Therefore, poor experiences of group relationships may actually harm players:

*There are times when it can create wounds instead, you get bad experiences because there’s bad people in every community …*

*(Stadler)*

This helps to acknowledge the reality that not all experiences of D&D communities will benefit players, whilst continuing to promote D&D as a meaningful strategy in creating and maintaining connections that may support coping for young adults.

#### 3.2.3. Remaining Present

The nature of collaborative storytelling in D&D helps players to remain present during gameplay. Whilst players may have their own character motivations and goals, the DM or other players can derail the best-laid plans by following a different narrative thread or revealing surprises to the adventuring party. Players spoke of the experience of tolerating the uncertainty provoked by the game mechanics, such as rolling dice to determine in-game outcomes, or cliffhangers between sessions:

*… you just cannot skip to the future, or … read a bit more or ask any further questions … you gotta wait for next session’.*

*(Pam)*

This lack of ability as both the character and player to determine what happens next evokes a sense of anxiety for some. However, for others, this uncertainty may evoke a sense of intrigue:

*I always really look forward to my characters failing because those are the really unpredictable moments and those are the moments where you really test the characters kind of limits.*

*(Florence)*

Additionally, players identified strategies for remaining present with their characters through creative means, such as dressing in clothing or listening to playlists inspired by their character. The sense of wonder promoted through these strategies appears to support players in learning to tolerate the unknown in game, raising a question as to how this may apply in a player’s reality.

### 3.3. D&D for Exploration

This theme looks at how the participants develop their self-knowledge through exploring aspects of their identity and societal roles through character creation and the facilitative role of roleplaying in this process.

#### 3.3.1. Identity-Affirming Play

The way in which players spoke about exploring their identity evoked varying levels of desire to use characters to actively explore parts of themselves, whether to replicate themselves as a character in game or to try out new or alternative aspects of identity:

*I always say that my characters are kind of like me with the dials toggled.*

*(Florence)*

This awareness of the multi-faceted nature of identity, as described by Florence, helps to explain how players engage in character creation and roleplay to explore elements of themselves, with some recognising difficulties in feeling a secure sense of self. Characters seemed to provide a layer of protection for players who did not feel ready to openly share parts of themselves as they navigated new social connections in young adulthood. Instead, players opted to use their character for “adopting a mask” (Bunny) or acting as a “filter” (Sable). The following quote describes the benefit of enacting parts of the player’s identity through their character:

*They don’t know like how much of what I’m saying is me. But there’s like some truth to it. And in in character form, they accept him and they love him … And it feels nice … and it’s like they don’t know me, but they do, but in a less scary way.*

*(Alexei)*

While the player recognises that their gaming peers do not know how much of them is present in their character, Alexei nonetheless took pleasure in the love and affection they received via their character’s interactions. However, once players developed trust in peers to be more accepting, they seemed to experience a greater sense of freedom to explore themselves openly:

*It allowed me that freedom to explore certain feelings and thoughts … parts of my identity that I’m now able to more freely embrace and accept.*

*(Bunny)*

This quote evokes the sense of celebration and joy players found in exploring themselves in a space where they felt a sense of belonging. Additionally, this seemed to provide a safer environment for players to explore minoritised aspects of their identity. Several players described the pivotal role their characters played in better understanding their gender identity:

*I was already kind of having issues and questioning my gender. And then in the first campaign I went, I’m going to play a boy … And then funnily enough, I really enjoyed using he/him pronouns and acting in a sort of masculine, typically masculine way.*

*(Nick)*

Whilst the player acknowledges that his journey with gender transition began before playing his D&D character, the gender euphoria and acceptance of peers experienced through his character’s he/him pronouns seemed to further validate his personal sense of gender identity. Furthermore, safer and inclusive D&D spaces can provide gameplay that players may use to explore their experience of difference. For example, Sable, who is the only Black player in their group, described their desire to challenge racial stereotypes through their character creation and roleplay:

*I’ve started coming around to it more often to play like sort, more, more either African or put in sort of my [redacted] heritage within the characters I play so they kind of either become like the preacher or a clergy member that I knew sort of. And and that’s quite fun to play in sort of extrapolate and see what that means.*

*(Sable)*

Here, Sable evokes a playfulness they have developed over time that appears to empower them to recognise the strengths in the intersectionality of their identity, seemingly reclaiming power over the narratives they and their character can tell.

#### 3.3.2. Social Roles

The collaborative nature of D&D, and the task of choosing a class for your character, appears to lend itself to players taking on a number of roles within their group:

*Are they a leader? Are they a supporter? Are they someone who is just there to get the job done?*

*(Ashe)*

Therefore, players appear to look beyond stereotyping of character class, for example, the cleric who can heal the party or the fighters to be up front in battle, instead considering a range of roles characters may embody. Some players acknowledged the reciprocal relationships between the characters they interact with and people they engage with outside of the game:

*Then the decision to kind of have her be older and have her own student … would be my way of … how I’ve since in recent years met other sort of neurodivergent folks in similar situations.*

*(Bunny)*

Here, Bunny describes her character taking on a teaching role that seems to reflect the experience she has in supporting neurodivergent peers new to engaging with D&D. Players making these links between themselves and their characters appeared to recognise their strengths, using D&D to explore the different ways to utilise these skills in their character roles and reality:

*It’s quite nice to be sort of like he was very much like the dad of the group … where they kind of can turn to you and ask for perspective, which is something that I sometimes do ….*

*(Sable)*

These experiences evoke a duty in becoming protectors in character, with several players likening this to being parent-like or to their role as an older sibling. In this example, James describes the simplicity of acting in line with this protector role through his character compared to how this may feel in reality:

*… in real life, it’s obviously usually a lot harder to try and work out what the right thing to do is, but for [my character] it tends to be quite simple … it’s kind of protect nature, protect friends.*

*(James)*

Each of these players evokes a sense of achievement and self-worth through having their characters align in a meaningful way with their values. The safety and connection experienced in these groups appears to promote the players’ wellbeing. However, as highlighted by Star, it is not always present in players’ realities:

*Western society is so individualistic that it is, you need an excuse to do collaborative things versus there are many other cultures and communities that are based off of communities of care and uh, collective activity and we’re kind of missing that in our society. So D&D is a way to build that in and then I think from like beyond D&D, it spreads outward.*

*(Star)*

Whilst Star expresses the challenges experienced by individualistic societies, the collectivist approach that players feel is promoted within D&D communities seems to increase hope that this may create a ripple effect that facilitates better collective care within wider society.

### 3.4. D&D for Growth

Lastly, D&D for growth describes the ways in which D&D provides a platform for young adults to practice skills when accessed in a safer environment where players have developed coping skills that provide opportunity to reflect and explore themselves. Players may then develop and apply these skills in their lives personally and occupationally, improving their confidence as they develop and grow as individuals.

#### 3.4.1. Self-Improvement

Despite the challenges of young adulthood reflected in the conscious drive to push out of their comfort zone in search of self-improvement, players who felt safer and supported by their group appeared to use their characters to complete experiments. For example, Nick reflected on testing out romantic scenarios through his character:

*… while that’s a foreign concept for me, it’s actually been quite fun playing it and going actually, no, I can see why people like this … And that’s really nice …*

*(Nick)*

By experimenting in this way, characters could be used to model potential outcomes, enabling better understanding of what might cause characters to fail or succeed, seemingly facilitating learning for the player about how to navigate similar scenarios in reality to achieve their desired outcome. The act of roleplaying and navigating these scenarios in character also develops the skills of the player, as noted here by Stadler:

*I work in a creative industry with creatives. And I now I understand a lot of how they think based on how I think playing D&D and how I sort of function and work. […] I was able to use a lot of those skills to help me finish my dissertation.*

*(Stadler)*

This exemplifies how the skills and understanding developed through the processes of playing D&D have practical applications for education and occupational purposes. This appears to hold value for the players, seemingly better supporting them in adapting to the changes of young adulthood.

However, even when characters may not have achieved the desired in-game outcomes, the nature of D&D seems to support characters, and therefore players, in finding alternative solutions. Sable shared the meaning they took from their experiences engaging with D&D in this way:

*It’s kind of allowed me to sort of look inward and go “look, you can live a life and just make mistakes … there is a chance that you might be able to go back and either rectify or make peace with them.*

*(Sable)*

Here, Sable described the acceptance their character has experienced in making mistakes whilst still succeeding more generally. This appears to enable the player to have compassion and hope for themselves as they continue working towards their goals.

#### 3.4.2. Confidence Boost

Despite some of the challenges that players may experience playing D&D, all players reported a positive impact on their wellbeing and mental health. From this, it appears that D&D enriches players’ sense of self, supporting them to continue engaging in their valued interests whilst adding to their skill sets for coping and daily living. Pam described a number of ways in which her improved confidence supports her:

*Me just having the confidence … can be applied in any kind of situation in this sort of scenario because either confidence in social situations, confidence when it comes to speaking up, also confidence to knowing that I can do more than I feel like.*

*(Pam)*

In this quote we can see how D&D provides opportunities which support personal and social development, amplifying the hopefulness characterised in the experiences of all participants who took part. The experiences presented by the players help to better understand the elements of D&D that protect and empower players whilst engaging safely.

## 4. Discussion

This study aimed to better understand how young adults explore their identity and mental health through playing D&D. The findings evoked four themes that reflect the experiences of the players interviewed, positioning D&D (1) as a safer space, (2) for coping, (3) for exploration, and (4) for growth. The themes suggest that the use of D&D evolves over time once players feel safer in the gameplay.

Players reported that D&D provided them with an opportunity to explore their identity when they had access to a safer D&D group. In one sense, this is somewhat at odds with theoretical models of identity formation that suggest that this occurs largely during adolescence (Erikson, 1950) and could add to the current critiques of this theory (e.g., Shanahan, 2000). Alternatively, this could reflect the further sense-making that occurs in early adulthood around family life cycles, work, and relationships (McGoldrick & Carter, 2003) or the “crises” that can occur at any point during the life cycle that create opportunities for continued identity development (Sokol, 2009). It may also be representative of a generational prolonging of the identity formation process due to the lost opportunities during the COVID-19 global pandemic (Khazvand et al., 2021). The players of the current study would have been between 13 and 20 years of age during the pandemic, meaning that they may have experienced these timeline shifts in exploring their social roles, navigating friendships, and balancing responsibilities. It is therefore understandable that players continued to explore their identity within the valued safer space that their D&D groups provided them with.

In seeking safety in their D&D group, players highlighted how their characters can provide a “mask” or “filter” in exploring newer or less certain parts of themselves personally and socially. The players in the current study describe masks as an opportunity to explore more freely when playing in a safer environment. The utility of roleplaying games to support the exploration of identity is well documented (Diakolambrianou & Bowman, 2023). These games provide an opportunity to test out different ways of being and problem-solve and can facilitate transformation of the self (Dennis, 2007). This process mirrors the roleplaying and externalising techniques used in psychological therapies, where challenges can be detached from identity and explored with a respectful and non-blaming, collaborative approach (Morgan, 2000; White & Epston, 1990). Specific roleplaying techniques such as chair work have been evidenced as effective for a range of mental health difficulties (Ottingerová et al., 2026). D&D may therefore provide another means by which to deliver experiential interventions.

Although the findings indicate players engaging in externalising and roleplay through D&D, this was not always the case. Instead, some experienced overlaps between their own and their characters’ emotional and relational experiences. This reflects previous findings exploring the concept of bleed (Bowman, 2023), which describes this flow of experiences from character to player and vice versa, whether these are helpful or unhelpful experiences (Causo & Quinlan, 2021). Bleed can describe the flow of skills and knowledge between players and characters and vice versa (Connell, 2023)—this is mirrored in the present sample, where bleed appeared to facilitate experiences of achievement, acceptance, and joy among players. However, it is important to hold in mind the potential negative impact of bleed that may exacerbate symptoms for players—especially for those who are already at higher risk of developing mental health difficulties (McGorry et al., 2022).

Finally, the themes and subthemes can be understood in relation to the Psychological Recovery Model (Andresen et al., 2003), as originally formulated by Causo and Quinlan (2021) when interviewing Australian adult players of D&D. The players shared experiences that align with the model’s concepts of finding hope, re-establishment of identity, finding meaning in life, and taking responsibility for recovery. The players interviewed here shared similar themes of identity exploration, hopefulness through their connection with others, personal growth, and exploring roles and the responsibilities these entail when navigating young adulthood. D&D spaces may therefore facilitate factors contributing to the promotion of wellbeing and mental health for young adults, with the current findings emphasising the importance of relational safety as a prerequisite.

### 4.1. Strengths and Limitations

Previous research in this area has presented the gender identity of participants in a binary way (e.g., Causo & Quinlan, 2021). A strength of this research is the high representation of those identifying as non-binary, many of whom shared the utility of D&D when exploring gender identity. This highlights the potential for D&D spaces as safer and supportive spaces in which to explore gender outside of binary concepts (e.g., Garcia, 2017; Gobble, 2021; Van Wert & Howansky, 2025).

We also had a majority representation of those identifying (either with or without a clinician’s diagnosis) as neurodivergent. D&D is popular within the neurodivergent community (Cross et al., 2023). Games more broadly are an engaging and effective means of supporting neurodivergent young people in developing skills and practicing them (Atherton & Cross, 2021). D&D specifically provides a platform to foster social communication skills as well as an opportunity to share a personal interest with others (Atherton et al., 2025). Akin to gender exploration, D&D may be particularly appealing and beneficial for those who are neurodivergent. The findings are limited to young people (aged 18–25). It is unclear if these themes, in particular those related to identity formation, are specific to this age group or may generalise to those who are older. Moreover, the small sample size restricts the breadth of experience voiced in the interviews. For example, the majority of players had experiences of higher education, whereas in the wider population only 35.8% of young people attend university at 18 years old (Bolton, 2024). Therefore, their experiences may differ from those of young adults on alternative pathways after leaving statutory education.

### 4.2. Clinical Implications

These findings add to the growing literature supporting the potential therapeutic benefits of D&D. Our findings add to this by identifying further potential mechanisms of change that could underpin the logic model for such TA-RPGs (Kilmer et al., 2023). Specifically, D&D could be a vehicle for delivering experiential therapies that align with the Psychological Recovery Model (Andresen et al., 2003). The process described by participants of exploring and growing in terms of their understanding of themselves aligns with Roger’s theory of the self (Moore & Oosthuizen, 2010), in particular the concepts of the ideal self and self-actualisation. D&D potentially provides a safer space where players can roleplay possible selves and practice these before making such changes in the real world. Further investigation of TA-RPGs is warranted.

### 4.3. Future Research

The findings here are relevant to players of D&D, but there is a need to understand the experience of DMs—particularly their experience of holding the D&D game space where players engage in identity exploration. For example, bleed is a present theme in our findings and the wider literature (Atherton et al., 2025; Connell, 2023) and guidance (Kilmer et al., 2023). It is unclear what impact this bleed may have on DMs’ wellbeing. Future research could explore this to provide insight for supporting DMs, whilst improving understanding for how practitioners may best be supervised if utilising D&D.

Lastly, the apparent overlap of how young adults are using D&D and aspects of psychological interventions warrants further exploration using robust methods. For example, randomised controlled trials evaluating TA-RPGs in terms of their effectiveness and acceptability are needed. If positive outcomes are found, D&D could offer an innovative and non-intimidating way of delivering psychological support within mental health and university wellbeing services.

## Figures and Tables

**Table 1:** Sample characteristics.

Characteristic	*n*	% of Total (*N* = 11)
Gender (*n*, %)		
Female	3	27.27
Male	3	27.27
Non-binary	5	45.45
Gender Same as at Birth		
Yes	5	45.45
No	6	54.55
Ethnicity		
Asian/Asian/British	2	18.18
White British	5	45.45
White Other	2	18.18
Mixed Ethnicity	2	18.18
Employment Status *		
Employed full-time (paid)	5	45.45
Employed part-time (paid)	3	27.27
Employed part-time (voluntary)	3	27.27
Student	5	45.45
Highest Level of Study		
Postgraduate taught	1	9.09
Sixth form/college	2	18.18
Undergraduate	8	72.73
Mental Health Diagnosis		
Yes—diagnosed by a health professional	6	54.55
No	5	45.45
Access to Mental Health Support		
Yes	9	81.82
No	2	18.18
Neurodevelopmental Condition Diagnosis		
Yes—diagnosed by a health professional	3	27.27
Yes—not diagnosed by a health professional	6	54.55
No	2	18.18
D&D Role		
Player only	6	54.55
Player & DM	5	45.45

Note. For characteristics marked with an *, participants could select multiple responses.

## Data Availability

The data presented in this study are available on request from the corresponding author.
